# Post-traumatic stress disorder following childbirth: a neglected cause

**DOI:** 10.3389/fgwh.2023.1273519

**Published:** 2023-12-07

**Authors:** Areeba Ahsan, Abdullah Nadeem, Ashna Habib, Areeba Aamir Ali Basaria, Rabeea Tariq, Nahid Raufi

**Affiliations:** ^1^ Department of Medicine, Dow University of Health Sciences, Karachi, Pakistan; ^2^ Department of Medicine, Kabul Medical University, Kabul, Afghanistan

**Keywords:** post-traumatic stress disorder, childbirth, neglected cause, low-income countries, mental health, healthcare, trauma-focused therapies

## Abstract

Post-traumatic stress disorder (PTSD) following childbirth is a significant mental health risk for women globally. However, it remains a neglected cause, particularly in low-income countries like Pakistan. This paper explores the unique challenges faced by women in such settings, including limited access to healthcare and social support, cultural norms, and stigma surrounding mental health. The diagnosis and causes of postpartum PTSD are discussed, along with its effects on both mothers and their infants. The lack of awareness and training among healthcare professionals in recognizing and treating postpartum PTSD is highlighted as a major barrier to adequate care. To address these challenges, the paper proposes a comprehensive approach, including raising public awareness, providing mental health support and resources, and integrating postpartum mental health into medical education. Trauma-focused therapies are recommended for effective treatment. The paper concludes with the need for more research in low-income countries and emphasizes the importance of understanding and supporting women with postpartum PTSD to improve their well-being and maternal and child health.

## Introduction

Post-traumatic stress disorder (PTSD) is a chronic mental condition that reduces one's quality of life and increases one's monetary burden. The development of PTSD is triggered by exposure to a traumatic stressor. A differentiation between ordinary and traumatic stresses (those with the potential to cause PTSD) is required for this. PTSD was initially described in the Diagnostic and Statistical Manual of Mental Disorders (DSM-III), and later modifications to the diagnostic criteria have been made. Traumatic stress is defined as the experience of actual or impending bodily harm, death, or sexual assault (1). PTSD refers to the complex physical, cognitive, emotional, and behavioral repercussions of psychological trauma. In comparison to other diseases, PTSD provides a challenging and distinct field for research, particularly in the postpartum period as this period marks a remarkable era in a woman's life, filled with various psychological, biological, and social changes. Women's mental health at this period can have serious consequences for the health of the mother, child, and family system, and posttraumatic stress disorder (PTSD) has been highlighted as a possible mental health risk for mothers. According to a recent meta-analysis, the mean prevalence of PTSD during the first postpartum year was 4.0% in community-based samples, with significantly higher rates (18.5%) in high-risk categories (e.g., mothers with pregnancy problems (2, 3). For women who are pregnant or have recently given birth, PTSD may pose a serious mental health risk (4). Globally, the incidence of PTSD is 3.9%, with delayed-onset PTSD accounting for 5.6% of cases, whereas prevalence rates at approximately 6 weeks postpartum ranged from 2.8 to 5.6% (5). Prenatal depression and anxiety, pre-pregnancy history of psychiatric problems, history of sexual trauma, childhood sexual abuse, intimate partner violence, psychosocial attributes, dread of labor, traumatic birth experiences, complications during pregnancy or childbirth, a lack of support, and dissociation during childbirth are just some of the elements that have been linked to the emergence of postpartum PTSD (1). Women with PTSD may experience symptoms such as sweating, nightmares, increased numbness, sudden recollections of distressing thoughts, avoiding birth-related reminders, anxiety, and despair, being easily startled, and feelings of hopelessness, dread, regret, or embarrassment in the weeks and months following childbirth (6). In low-income countries like Pakistan, where access to high-quality prenatal healthcare and mental health services may be limited, the challenges experienced by women with Postpartum PTSD are particularly severe. Here, we look at the unique experiences and difficulties that women of low-income nations encounter when dealing with Postpartum PTSD in order to provide insight on the complex interaction of cultural, sociological, and economic elements that contribute to their higher sensitivity to the disease. Understanding the unique challenges faced by women in low-income countries may help with policy reforms and targeted initiatives to improve the care and well-being of new mothers in developing countries.

## Diagnosis and causes of post-traumatic stress disorder

A clinical diagnosis of PTSD can be made by using the Clinician-Administered PTSD Scale (CAPS), a 17-item structured interview based on DSM-IV-TR criteria. The F1/I2 item rule is used, according to which a symptom is regarded as endorsed if both its reported frequency and intensity are greater than 1. According to DSM-IV-TR, at least one intrusion symptom, three avoidance symptoms, and two hyperarousal symptoms should be present to diagnose PTSD. Concerning demographics, the CAPS has outstanding reliability, convergent and discriminant validity, diagnostic value, and sensitivity to clinical change (7). Modified PTSD Symptom Scale (MPSS-SR), PTSD Symptom Scale Interview (PSS-I and PSS-I-5), PTSD Checklist for DSM-5 (PCL-5), PTSD Symptom Scale Self-Report Version (PSS-SR), and Short PTSD Rating Interview (SPRINT) are some of the scales to determine PTSD, and each instrument offers evidence of reliability and validity; even some are free of charge (8). However, in low-income nations like Pakistan, diagnosing postpartum posttraumatic stress disorder (PPTSD) can be difficult due to a number of factors, including limited resources, cultural norms, and the stigma associated with mental health conditions. The Clinician-Administered PTSD Scale (CAPS) and the Mississippi Scale for Combat-Related PTSD (MPSS-SR) are not frequently used in the Pakistani context of postpartum PTSD assessment.

The causes of postpartum PTSD are not fully understood, but they are thought to be linked to a mix of biological, psychological, and social factors. A lengthy or traumatic birth experience with any unexpected complications can trigger PTSD. Poor relationships with the baby's father, a lack of social support from family and friends, financial issues, and difficulty adjusting to motherhood can also contribute to PTSD. Complications and traumatic labours are more likely when prenatal care is insufficient and deliveries are performed by untrained workers. In addition, a lack of resources and social expectations may restrict women's ability to make choices during childbirth, resulting in the helplessness and powerlessness that are linked to Postpartum PTSD. If left untreated, the condition can cause severe psychological distress and negative health outcomes.

## Current challenges

Postpartum PTSD can have a wide range of effects on both the mother and the child. For the mother, it can cause anxiety, depression, and isolation, as well as difficulties bonding with the baby. For the child, it can lead to poor nutrition, due to the mother's difficulty or inability to breastfeed. A longitudinal study (9) revealed that postpartum PTSD in mothers is significantly associated with an inability to initiate breastfeeding, which can have serious implications for the health of both the mother and the child. The difficulties may include difficulty producing milk, latching the baby onto the breast, and maintaining an adequate milk supply, all of which can result in poor nutrition for the child. In addition, postpartum PTSD can cause depression and anxiety that can prevent the mother from effectively parenting and forming secure bonds and attachment relationships with the child, which are essential for the child's future positive development (10).

Postpartum PTSD is a significant yet sometimes disregarded factor. Although there has been progress, it is still frequently undiagnosed and untreated, and the majority of healthcare professionals lack the necessary training. This implies that many women are left without the support they require because the majority of psychological and emotional therapy offered by child health care agencies does not even address postpartum PTSD. Many people are also ignorant of the existence of this illness. Numerous moms have been unable to get the assistance they need due to a lack of acknowledgment and empathy, forcing them to suffer alone. Additionally, healthcare personnel are unprepared to recognize and manage postpartum PTSD. To address this, childbirth delivery services must recognize it as a serious mental health condition and prioritise it. They must also take into account the psychological and emotional needs of mothers suffering from it and ensure they get the care and support they need to manage the condition.

Many women experience a certain degree of apprehension when considering a subsequent pregnancy, particularly if their prior birth or postpartum recovery was complicated. This fear can result from numerous factors, such as feeling overwhelmed and unable to cope with another pregnancy, the potential complications, the physical and emotional toll, and the financial burden. For some, the fear is intense enough to lead to the avoidance of reminders, like doctor's appointments and conversations about having another baby.

In low-income countries like Pakistan, postpartum PTSD is often overlooked, and women are expected to quickly return to their daily lives with few or no resources to turn to for help. Cultural and social factors influence how women and their families perceive, interpret, and cope with PTSD in Pakistan. Thirteen significant predictors of PPD were established in a meta-analysis of 84 studies conducted during the 1990s (11). That is, prenatal depression, self-esteem issues, childcare stress, prenatal anxiety, life stress, social support, marital relationship, history of prior depression, infant temperament, the maternity blues, marital status, socioeconomic status, and unintended or unwanted pregnancies in descending order of importance. In developed nations, these issues may be rectified, but in developing countries like Pakistan, they persist and are the root of post-traumatic stress disorder. It has been demonstrated that sociodemographic factors influence both PTSD and pain, with poorer socioeconomic position, measured by income, work status, educational attainment, and neighbourhood poverty level, associated with more severe PTSD symptoms and worse pain outcomes (12, 13). In developing countries like Pakistan with low literacy rates, symptoms of PTSD may not be recognized by many women and their families as a mental health issue, which can result in underreporting and poor or delayed treatment. Additionally, due to the stigma associated with mental health conditions, women with PTSD may be reluctant to seek assistance or freely communicate their experiences out of fear of being judged or disgraced by their relatives and communities. Family support can be helpful, but it may also prevent someone from getting professional assistance. Family members may dissuade women from obtaining medical or psychological care if they think postpartum distress is a natural part of the process. Instead of pursuing empirically supported mental health care, some women may turn to alternative healers or cures. As a result, the condition is often left untreated, which can lead to further complications. In Pakistan, traditional gender roles may assign women certain roles and responsibilities during and after childbirth. It may be difficult for women to express their traumatising sentiments or seek treatment, which increases their stress and emotional load. Obstetricians and midwives in Pakistan may not have the necessary training to screen for PTSD or recognize mental health conditions in new moms. Women from low-income backgrounds may have a harder time finding appropriate PTSD treatment. Furthermore, supporting mothers with postpartum PTSD is a major challenge in low-income countries. One of the main reasons is the lack of access to and availability of mental health care in these countries. The healthcare system lacks resources, staff, and qualified mental health professionals. Mental health is often neglected in low-income countries, leading to a lack of awareness and understanding of postpartum PTSD among the general public and healthcare professionals. Consequently, effective screening, diagnosis, and treatment of postpartum PTSD are not supported by substantial financial resources.

A cross-sectional study (14) has determined that postpartum PTSD is a serious issue that needs to be addressed as it can harm women's welfare, mother-infant attachment, and child development. To do so effectively, the best strategy in a developing nation like Pakistan is to start by raising awareness. If postpartum posttraumatic stress disorder is ignored in underserved parts of Pakistan or any other country, it can have serious ramifications for the affected women, their families, and the community. Without the necessary help and acknowledgement, women who are experiencing Postpartum PTSD can continue to suffer in silence. Postpartum PTSD can hinder a mother's capacity to form a strong bond with her newborn, which makes it more difficult to do so. For the child's long-term emotional and psychological growth, this may have negative effects. Postpartum PTSD, left untreated, can cause conflicts within the family. The woman's mental suffering can cause disagreements and communication problems with her partner, which would change the dynamics of the family. Postpartum PTSD left untreated can have a substantial negative impact on a woman's capacity to carry out daily tasks, such as caring for her family and going to work. Suicidal ideation, which refers to thoughts of self-harm or suicide, can occasionally result from Postpartum PTSD symptoms that are particularly severe. It's critical to spot the warning symptoms of suicidal ideation and support Postpartum PTSD-affected women by taking the right steps. By developing public health campaigns specifically focused on postpartum PTSD to raise awareness among healthcare providers and the general public through community outreach, such efforts can help women, families, and healthcare providers recognize the signs and symptoms of postpartum PTSD. Increased awareness and support are key to helping to provide the care that these women require.

## Efforts and recommendations

Healthcare providers should be trained to recognize and accurately evaluate the signs and symptoms of postpartum PTSD and administer appropriate treatment which can include a multifaceted approach. Healthcare providers should provide accurate information about PTSD, its symptoms, and the commonality of postpartum PTSD experiences. Normalizing these feelings can alleviate anxiety and promote understanding. In some cases, medication, such as antidepressants or anti-anxiety drugs, might be prescribed to manage severe symptoms. Providers can educate families about PTSD, enabling them to provide understanding and encouragement as emotional support from healthcare providers, family, and friends is invaluable. Additionally, referral to specialized mental health professionals is essential. These experts can conduct comprehensive assessments and provide evidence-based therapies (described below), tailored to individual needs. These trauma-focused therapies can help modify thought patterns, process traumatic memories, and reduce anxiety and avoidance behaviours.

Midwives who are deeply involved in the childbirth process, play a pivotal role in supporting individuals dealing with postpartum PTSD in resource-limited settings. Their continuous presence before, during, and after childbirth allows for early detection of distress signs. In addition to their medical role, midwives offer emotional support, acting as compassionate listeners during a vulnerable time. When signs of postpartum PTSD are identified, midwives can offer immediate comfort, encouraging affected individuals to seek professional help. Their knowledge of the local community dynamics also enables them to tailor their assistance effectively, ensuring that individuals affected by postpartum PTSD receive comprehensive and culturally sensitive support.

A timely diagnosis might help in identifying the early indicators of more severe mental illnesses like stress, anxiety, depression, and other behavioural disorders and can prevent the symptoms from getting worse.

To do so, the government can partner up with international organisations that specialise in mental health to provide training and resources to healthcare providers, allocate funding for mental health services and resources in maternal healthcare facilities, create policies and guidelines for maternal healthcare providers to identify and manage postpartum PTSD, and incorporate postpartum mental health into medical and nursing school curricula to ensure that healthcare providers are knowledgeable about postpartum PTSD.

Develop educational materials and programs for expectant mothers and their families to increase awareness of postpartum PTSD and the importance of seeking help. Providing support and education to husbands and family members can help them understand the impact of traumatic childbirth and how they can support the women in their lives. This can include education on PTSD symptoms, providing emotional support, and helping with household tasks and childcare.

Health services must be made available and accessible. Ensuring that women have access to mental health services, such as counselling and medication management, can help reduce the impact of PTSD following childbirth. This can include providing referrals to mental health professionals and ensuring that mental health services are covered by insurance.

In resource-limited settings, the involvement of communities and families is pivotal. Community leaders and local organizations can initiate awareness campaigns, dispelling myths and reducing the stigma surrounding mental health issues. Families, especially partners and close relatives, play a vital role in supporting individuals with postpartum PTSD. They can offer emotional support, assist with tasks, and provide a listening ear, easing the burden on the affected person. Educating families about the condition is crucial, including its symptoms and the significance of professional help. Partners' involvement in therapy or support groups enhances family unity and understanding. In therapy sessions, family participation provides valuable insights for therapists, enabling targeted guidance. Open communication within families fosters trust and emotional safety, essential for the healing process. Providing psychological support to women who have experienced traumatic childbirth can help reduce the risk of PTSD. This can include individual counselling offering Birth reflection and debriefing services, where women can discuss their childbirth, or group therapy and support groups that bring together women with comparable experiences to share and listen to each other's experiences to make them understand that they are not alone and that many mothers experience similar things. With the right support and treatment, the effects of PTSD can be mitigated, and mothers can be empowered to provide their children with the care and support they need and deserve.

On the other hand, additional approaches like Trauma-focused therapies should be encouraged and practised as they are evidence-based and effective in treating and addressing the psychological and emotional impact of traumatic experiences (15).

Some examples of trauma-focused therapies include:

• **Cognitive Behavioural Therapy (CBT):** CBT helps individuals identify and challenge negative thoughts and beliefs related to their traumatic experiences. This therapy can help reduce symptoms such as anxiety, depression, and PTSD (16).
• **Eye Movement Desensitization and Reprocessing (EMDR):** EMDR is a type of therapy that uses eye movements or other forms of rhythmic, bilateral stimulation to help individuals process traumatic memories and reduce distressing symptoms (17).
• **Prolonged Exposure Therapy (PE):** PE is a therapy that involves gradually exposing individuals to their traumatic memories in a safe and controlled environment. Through repeated exposure, individuals can learn to face and process their traumatic experiences and reduce their symptoms (18).
• **Narrative Exposure Therapy (NET):** NET is a therapy that involves helping individuals create a coherent narrative of their traumatic experiences. This therapy can help individuals integrate their experiences into their lives and reduce distressing symptoms (19).

Society also needs to become more accepting of mental health issues and more supportive of those affected. Several steps need to be taken in Pakistani society to promote understanding and acceptance of mental health disorders, including postpartum posttraumatic stress disorder. To educate the public on mental health, public awareness initiatives should be started. To reach a wide audience, these efforts can work with local influencers and leaders as well as on a variety of media channels. Destigmatizing mental health issues ought to be top of mind. Open communication about mental health in the home, at school, and in religious institutions can help dispel misconceptions and promote empathy and understanding. By including mental health education in the school curriculum, we may encourage a generation that is more accepting and understanding of people who are dealing with mental health issues. By applying these strategies, Pakistani society can make tremendous strides toward acknowledging mental health conditions such as Postpartum PTSD and giving much-needed support and care to individuals affected.

Additionally, in developing nations like Pakistan, there is still a lack of research on postpartum posttraumatic stress disorder. A thorough understanding of the prevalence, risk factors, and efficient therapies for Postpartum PTSD in such settings is hampered by a shortage of studies in the area. Future studies should concentrate on carrying out extensive epidemiological investigations to ascertain the prevalence of postpartum PTSD in various regions of Pakistan and close this gap. Postpartum PTSD is influenced by several cultural and societal elements in the country, and a qualitative study examining the lived experiences of women who have undergone or suffered traumatic childbirth or postpartum events might offer important insights. Furthermore, longitudinal research tracing the evolution of Postpartum PTSD symptoms over time can provide insight into their long-term effects on women's mental health.

## Barriers to treatment in low-income countries

In low-income countries like Pakistan, there are several barriers to the identification and treatment of postpartum PTSD. These barriers often prevent women from accessing the care they need and exacerbate the impact of the condition on their mental health and well-being.

One of the significant challenges in low-income countries is the scarcity of mental health resources and facilities. There is a shortage of qualified mental health professionals and a lack of specialized services for postpartum mental health. This scarcity of resources makes it difficult for women with postpartum PTSD to access appropriate treatment and support. Mental health issues, including postpartum PTSD, are often stigmatized in many cultures. Women may hesitate to seek help due to fear of being judged or ostracized by their families and communities. Stigma can also lead to a lack of understanding and empathy from family members, preventing women from receiving the necessary support and encouragement to seek treatment.

In low-income countries, there is often limited awareness and understanding of postpartum PTSD among the general public and healthcare professionals. Many women and their families may not recognize the symptoms of PTSD as a mental health issue, leading to underreporting and delayed or inadequate treatment. Socioeconomic factors, such as poverty, lack of education, and limited access to healthcare, can also contribute to the challenges of managing postpartum PTSD. Women from low-income backgrounds may have a harder time finding appropriate mental health services and may face additional stressors that exacerbate their symptoms.

Traditional gender roles and expectations in some cultures may limit women's ability to express their traumatic experiences or seek help. The pressure to conform to societal expectations of being a stoic and resilient mother can prevent women from acknowledging their distress and seeking assistance.

## Recommendations for overcoming barriers

To address the barriers and challenges faced by women with postpartum PTSD in low-income countries like Pakistan, several strategies can be implemented:

Healthcare providers should receive training in recognizing and assessing postpartum PTSD symptoms. A healthcare provider includes obstetricians, nurses, midwives, and community health workers. Their training should focus on accurately identifying and evaluating the signs and symptoms of postpartum PTSD. This entails understanding manifestations such as flashbacks, anxiety, and mood disturbances related to traumatic childbirth experiences. The training program should emphasize basic counselling skills, active listening techniques, and the ability to create a supportive and empathetic environment. Integrating psychoeducation into the training is crucial. This training can help improve early identification and intervention, ensuring that women receive the support they need. Furthermore, clear referral protocols must be established to guide healthcare providers in connecting affected individuals with appropriate mental health resources. Public health campaigns specifically focused on postpartum PTSD should be developed to raise awareness among healthcare providers and the general public. These campaigns can educate people about the signs and symptoms of postpartum PTSD and the importance of seeking help.

Expecting healthcare providers to manage their responsibilities without necessary mental health resources is indeed unrealistic. In these contexts, basic training can be provided to healthcare providers, enabling them to identify PTSD necessary symptoms and offer initial support. Task shifting, involving non-specialized healthcare workers like community health workers, can bridge the gap by providing preliminary psychological aid and referral assistance. Telehealth services staffed by mental health professionals can offer remote access to expertise.

Mental health services should be integrated into maternal healthcare facilities, making them more accessible to women. This includes providing referrals to mental health professionals and ensuring that mental health services are covered by insurance. Educational programs and support groups should be established for expectant mothers and their families. These initiatives can help increase awareness of postpartum PTSD and provide emotional support to women and their families. Local organizations can raise awareness about postpartum PTSD through community campaigns, reducing stigma, and promoting understanding. These organizations act as intermediaries, linking individuals with available mental health resources and therapy services. Crisis hotlines staffed by mental health professionals provide immediate assistance.

More research on postpartum PTSD in low-income countries is essential to understand the prevalence, risk factors, and effective treatments. Longitudinal studies can provide valuable insights into the long-term impact of postpartum PTSD on women's mental health. Efforts should be made to destigmatize mental health issues in society. Open communication about mental health in schools, homes, and religious institutions can help dispel misconceptions and promote empathy and understanding (Table 1).

**Table 1:** Challenges and comprehensive recommendations for addressing postpartum post-traumatic stress disorder (PTSD) in low-income countries like Pakistan.

Challenges	Comprehensive recommendations
Limited access to healthcare	- Establish mental health resources and services in maternal healthcare facilities.
	- Train healthcare providers, including midwives and obstetricians, to recognize and assess postpartum PTSD symptoms.
	- Ensure mental health services are affordable and accessible to all women.
Cultural norms and stigma	- Develop culturally sensitive public health campaigns to raise awareness and reduce stigma.
	- Work with community leaders and influencers to promote understanding and acceptance of mental health issues.
	- Incorporate mental health education into school curricula to combat misconceptions.
Lack of awareness and understanding	- Conduct research to understand the prevalence, risk factors, and effective treatments for postpartum PTSD.
	- Implement public awareness initiatives through media channels and community outreach programs.
	- Establish support groups and educational programs for expectant mothers and families.
Socioeconomic factors	- Integrate mental health services into maternal healthcare facilities and subsidize mental health treatments for low-income women.
	- Provide financial assistance or insurance coverage for mental health services.
Limited resources and facilities	- Partner with international organizations to provide training and resources for mental health professionals.
	- Allocate funding for mental health services and resources in maternal healthcare facilities.
Traditional gender roles and expectations	- Conduct educational programs to challenge traditional gender roles and encourage open communication about mental health.
	- Involve husbands and family members in the education and support process.
Inadequate training for healthcare providers	- Integrate postpartum mental health into medical and nursing school curricula.
	- Provide ongoing training and workshops for healthcare providers to improve their knowledge and skills in recognizing and treating postpartum PTSD.

## Conclusion

Postpartum PTSD is a significant mental health issue that can have profound effects on women's well-being and the development of their children. In low-income countries like Pakistan, the challenges faced by women with postpartum PTSD are particularly severe due to limited resources, cultural norms, and stigma. Efforts to raise awareness, improve access to mental health care, and promote understanding and acceptance of mental health issues are critical in addressing these challenges and providing the care and support that women with postpartum PTSD need to recover and thrive. By implementing these recommendations and investing in mental health resources, policymakers and healthcare providers can work towards improving the well-being of new mothers in developing countries and create a more supportive and inclusive environment for those affected by postpartum PTSD.

## Data Availability

The original contributions presented in the study are included in the article/Supplementary Material, further inquiries can be directed to the corresponding author.
